# Clinical Success of Biophoton Therapy in Improving Cognition of Alzheimer’s Disease

**DOI:** 10.1002/alz.094178

**Published:** 2025-01-09

**Authors:** James Z Liu

**Affiliations:** ^1^ First Institute of All Medicines, Milford, DE USA

## Abstract

**Background:**

Alzheimer’s disease (AD) poses a significant global health challenge, which demands innovative therapeutic approaches. The strong biophoton field generated by breakthrough medical devices has preliminarily shown a high rate of success in improving cognition and life quality of the patients with AD.

**Method:**

This was a randomized triple blinded placebo controlled clinical study to verify if biophotons can improve cognition of AD. The clinical trial was to enroll a total of 30 participants diagnosed with moderate to severe AD. All participants continued their standard care if any.

**Result:**

During a 4‐week observational period, 16 participants in the treatment group used the biophoton generators daily. Cognitive function, life quality and EEG were assessed using standardized measurements. Additionally, bioenergy of brain was determined using a non‐linear bioenergy scanner. Preliminary analysis reveals a remarkable improvement in cognitive performance among participants in the biophoton therapy group compared to the control group. Seventy‐five percent (75%) participants reduced the Alzheimer’s Questionnaire severity; 88% increased life quality SF‐36 score; and 94% increased brain bioenergy. All were significantly different from the Control group (P < 0.01). EEG test also showed that the “missed response” was reduced in the Treatment group. Caregivers reduced the stress and difficulty in caring for their seniors. No adverse effects were observed.

**Conclusion:**

These promising results highlight the potential of biophoton therapy as a novel and safe intervention for Alzheimer’s disease. Further investigations are warranted to elucidate the underlying mechanisms. This clinical study represents a significant step forward in treating Alzheimer’s disease.

